# Homocysteine induces ferroptosis in endothelial cells through the systemXc^−^/GPX4 signaling pathway

**DOI:** 10.1186/s12872-023-03342-4

**Published:** 2023-06-24

**Authors:** Jiahao Shi, Di Chen, Zilin Wang, Shaolin Li, Shuying Zhang

**Affiliations:** Zhongshan Hospital of Dalian University, Dalian, China

**Keywords:** Ferroptosis, Vascular endothelial cells, SystemXc^−^/GPX4 signaling pathway, Homocysteine

## Abstract

**Objectives:**

To investigate whether ferroptosis is involved in HCY-induced endothelial injury and the possible mechanism of HCY-induced ferroptosis.

**Methods:**

EA. hy926 cells were cultured in vitro. Cells were intervened using HCY and Fer-1. The cells were divided into Control groups, HCY (4 mM), HCY (8 mM), HCY + Fer-1 (4 mM HCY + 0.5/2.5/5 µM Fer-1). CCK-8 assay was used to detect cell viability; Flow Cytometry was used to detect cellular Lip-ROS, TBA and Microplate assay was used to detect MDA&GSH, Western blot was used to detect the expression of ferroptosis-related proteins GPX4 and SLC7A11.

**Results:**

HCY can inhibited the proliferation of EA. hy926 cells in a time- and concentration-dependent manner; Fer-1 inhibits HCY-induced ferroptosis in EA.hy926 cells in a concentration-dependent manner; Compared with the control group, the cell viability and GSH content in the HCY group was significantly decreased (p < 0.05), and the Lip-ROS and MDA were significantly increased (p < 0.05); After co-culture of HCY and Fer-1, compared with the HCY (4 mM) group, the cell viability and GSH content in the co-culture group were significantly increased (p < 0.05), and the Lip-ROS and MDA were significantly decreased (p < 0.05) in a concentration-dependent manner; Western blotting results showed that the protein expression levels of ferroptosis-related proteins GPX4 and SLC7A11 in each experimental were significantly decreased after HCY treatment (p < 0.05), and Fer-1 could significantly reverse this effect.

**Conclusions:**

(1) HCY can induce ferroptosis in vascular endothelial cells. (2) HCY may induce vascular endothelial cell ferroptosis through the system Xc–GSH-GPX4 signaling pathway.

**Supplementary Information:**

The online version contains supplementary material available at 10.1186/s12872-023-03342-4.

## Introduction

Nowadays, the incidence of coronary atherosclerotic heart disease remains high, of which atherosclerosis (AS) is the basic cause. The formation of atherosclerosis is a progressive pathological change in which multiple factors stimulate blood vessels for a long time, of which endothelial dysfunction and vascular endothelial cell damage and death are the first changes of atherosclerosis, which is closely related to atherosclerotic plaque formation [1]. Therefore, effectively reducing the occurrence of endothelial injury is currently a research hotspot for atherosclerotic diseases.

A novel mode of cell death with iron ion-dependent, the oxidized form, ferroptosis (Ferroptosis), was first discovered and reported by Dixon [2] in 2012. It is a form of death characterized by abnormal intracellular iron metabolism, dysfunction of the glutathione-dependent antioxidant system, and massive accumulation of lipid hydroperoxides. Numerous studies have found that the chain reaction axis represented by the SystemXc^−^-GSH-GPX4 system is one of the most classical pathways of ferroptosis [2]. At present, ferroptosis is involved in the fields of the tumor, neurological degeneration, ischemia-reperfusion, etc., but the specific mechanism of ferroptosis in the pathological process of atherosclerotic diseases has not been concluded, which needs to be confirmed by more experimental data. Homocysteine (HCY) accelerates the formation of sclerotic plaques in arteries [3, 4], but whether it can cause endothelial cell injury through the ferroptosis pathway is unknown.

**Fig. 1:**
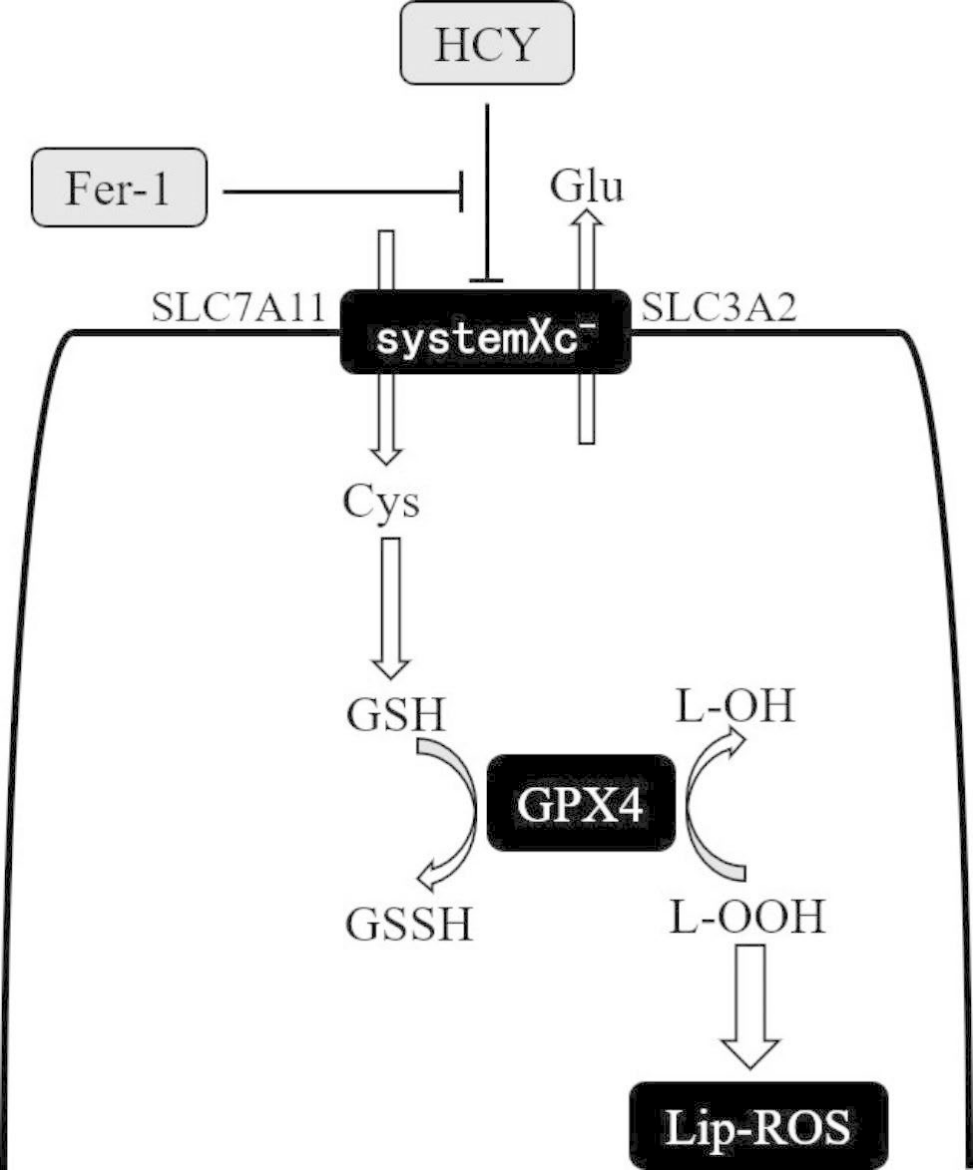
Pathways of SystemXc^−^/GPX4

Based on the current research status, this study intends to investigate whether homocysteine can play a role in vascular endothelial cell injury through the ferroptosis pathway and whether it mediates ferroptosis in cells through the classical pathway SystemXc^−^/GPX4 axis (Fig. 1).

## Materials and methods

### Main reagents and instruments

DL-homocysteine (Abcam, USA); Ferrostatin-1 (Abcam, USA); BODIPY ™ 581/591 C11 (Thermo Fisher, USA); Cellular MDA Assay Kit (Jiancheng, Nanjing, China); GSH Assay Kit (Jiancheng, Nanjing, China); BCA Protein Quantification Kit (Kaiji Biological, China); GPX4 antibody (Wuhan Sanying, China); SLC7A11 antibody (Abcam, USA); GAPDH antibody (Wuhan Sanying, China); HRP-Goat anti-mouse antibody (Wuhan Sanying, China); Light microscope (Nikon, Japan) 5% CO2 constant temperature incubator (SHELL, USA); Flow cytometer (FC-500) (BD, USA); multi-functional microplate reader (Thermo Fisher, USA); BIO-RAD electrophoresis apparatus and gel imager (VDS) (BIO-RAD, USA).

### Cell culture

The EA. hy926 cells were purchased from the Cell Bank of the Chinese Academy of Sciences. DMEM/F12 complete medium containing 10% fetal bovine serum and 1% penicillin-streptomycin mixture was prepared; After recovery and passage, EA. hy926 cells were cultured in a 37℃, 5% CO2 constant temperature incubator until the cell proliferation density reached more than 90% for the experiment.

### Experimental grouping

According to the cytotoxicity test results of HCY and Fer-1 before the experiment, two concentrations of HCY at high and low (4, 8 mM) and three concentrations of Fer-1 at low, medium and high (0.5, 2.5, 5 μm) were selected for experimental grouping. The cells were divided into Control groups, HCY (4 mM), HCY (8 mM), HCY + Low concentration Fer-1 (4 mM HCY + 0.5 µM Fer-1), HCY + Medium concentration Fer-1 (4 mM HCY + 2.5 µM Fer-1), HCY + High concentration Fer-1 (4 mM HCY + 5 µM Fer-1).

### CCK-8 assay

The cells with good growth status and proliferation density meeting the experimental criteria were digested, centrifuged and then the cells were counted and inoculated into each well of a 96-well culture plate at an appropriate density. After the cells adhered, the cells in each experimental well were treated with drug intervention according to different groups and cultured in a 37℃, 5% CO2 incubator for 22 h. At the end of the intervention, each experimental well was rinsed using PBS and cultured in a serum-free medium containing 10% CCK-8 for 2 h in the dark. The absorbance value at 450 nm was measured using a microplate reader and the cell survival rate was calculated.

### Lip-ROS content by Flow Cytometry

The cells with appropriate growth density were seeded in six-well plates, and the cells were treated with drug intervention for 24 h according to different groups. At the end of stimulation, remove the culture medium in each well, wash with PBS solution to remove the drug residue in each well, add BODIPYTM 581/591 C11 solution at the final concentration of 10 μm to each well, and incubate in a constant temperature incubator for 30 min, protected from light. PBS was used to fully wash off the dye and add an appropriate amount of trypsin for digestion, followed by centrifugation at 4℃ for 5 min at a speed of 3000 r/min. Remove the cell supernatant, add an appropriate amount of serum-free medium to mix the cells and centrifuge again, resuspend cells in 500 ul PBS and transfer to flow tube, and the fluorescence intensity was detected by FL2 channel.

### Determination of intracellular GSH and MDA content

The cells in each well of the six-well plate were treated with drug addition for 24 h according to different groups. Add an appropriate amount of precooled PBS to rinse the cells, use a cell scraper to scrape the cells and collect them into an EP tube, and perform centrifugation at 3000 r/min for 5 min at 4℃, remove the cell supernatant and add precooled PBS to resuspend the cells. The cells were ground and broken by the ultrasonic disruption instrument, centrifuged again for 20 min and then the supernatant was taken for detection according to the instructions for use of MDA and GSH reagents.

### Western blot

The cells in the culture dish were subjected to drug intervention for 24 h according to different groups. An appropriate amount of cell lysate was added and lysed on ice for 30 min. After collecting the cells, the cells were centrifuged at 13,000 r/min for 20 min at 4 ℃. The supernatant was taken for concentration quantification according to the BCA protein kit instructions. Protein was separated by SDS-PAGE (10%) with high-temperature denaturation, 400 A constant flow membrane, 5% skimmed milk powder for 2 h, primary antibody (GPX4 1:5000, SLC7A11 1:5000, GAPDH 1:10000) incubation overnight at 4 ℃, PBS rinsing for 3 times, secondary antibody (1:5000) was added to shake the membrane for 1 h, PBS rinsing for 3 times, ECL luminescence solution was added dropwise for protein development and ImageJ was used for grayscale analysis.

### Statistical analysis

All data are shown as mean ± standard error of the mean (SEM). All statistical analysis was performed using SPSS or GraphPad Prism 8.0. Levene test was performed to assess the equality of variance. Unpaired Student t-test was done to compare two groups. One-way ANOVA with Bonferroni correction for multiple comparisons was used to compare more than two groups. Statistical significance was set at p < 0.05.

## Results

### Fer-1 inhibits HCY-induced cellular ferroptosis

**Fig. 2.1:**
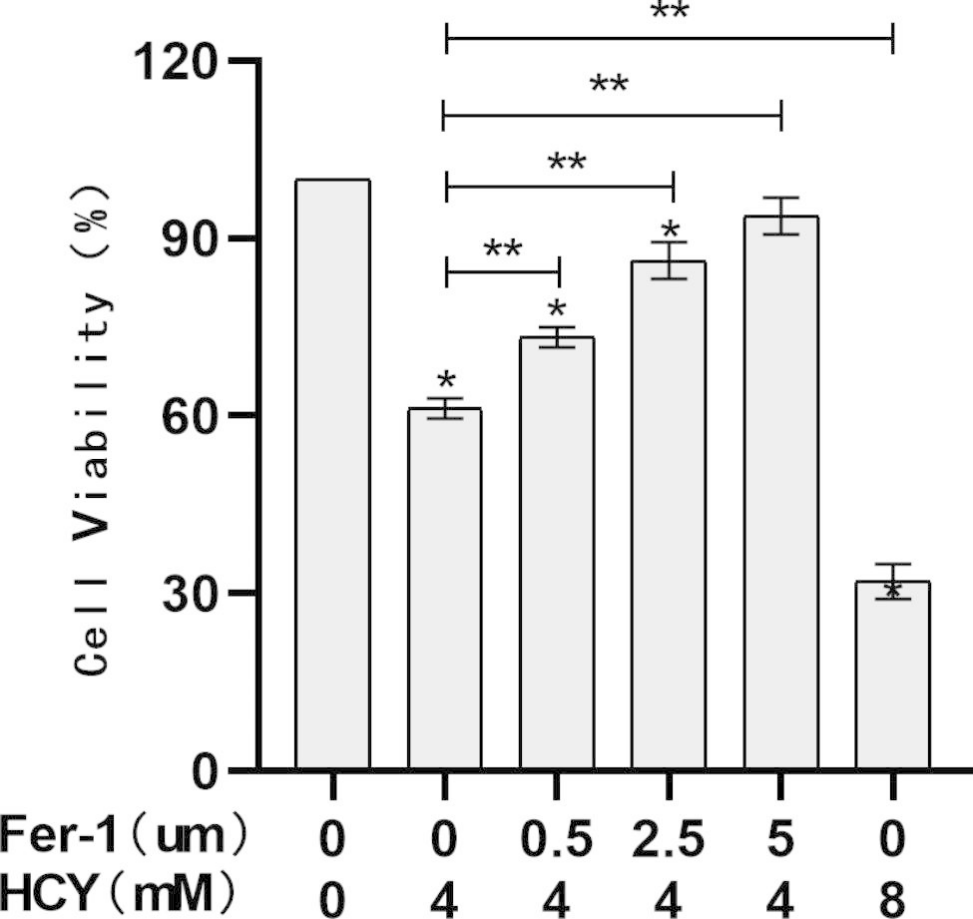
Effect of Fer-1 on HCY induced EA.hy926 cell viability(‾x±s, n = 3) * denotes *p <* 0.05 compared with control group; ** denotes *p <* 0.05 compared with HCY (4 mM) group

Compared with the control group, the survival rate of EA. hy926 cells decreased with the increase of HCY drug concentration (*p < 0.05); Fer-1, an ferroptosis inhibitor, reversed the effect of HCY on the effect of EA. hy926 cells, indicating that ferroptosis inhibitors could ameliorate the effect of HCY on the damaging effect of EA. hy926 cells, and the survival rate of cells increased in a gradient with the increase of Fer-1 concentration (**p < 0.05) (Fig. 2.1).

### HCY Induced cell lipid peroxidation injury

**Fig. 2.2:**
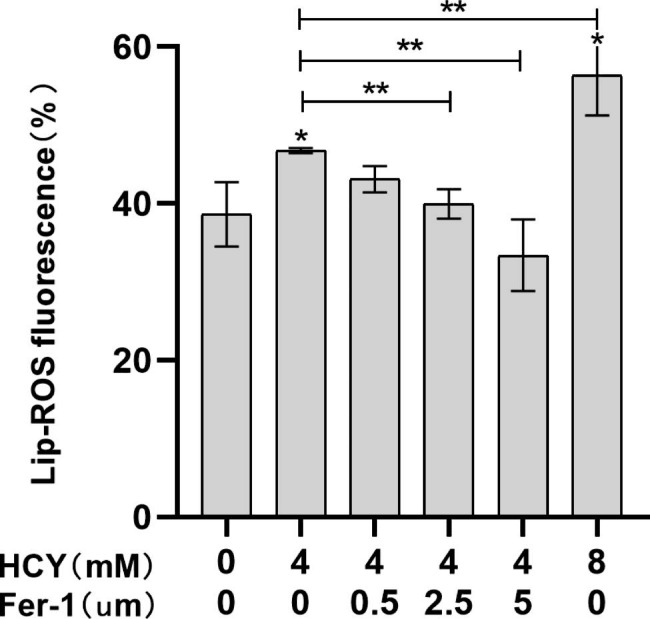
Lip-ROS accumulation in EA.hy926 cells after HCY treatment(‾x±s, n = 3) * denotes *p <* 0.05 compared with control group; ** denotes *p <* 0.05 compared with HCY (4 mM) group

The intracellular Lip-ROS level increased after HCY treatment, with statistical significance compared with the control group (*p < 0.05) (Fig. 2.2.A&B), and the tendency of lipid peroxidation was more significant with the increase of HCY concentration (**p < 0.05); while the Lip-ROS content gradually decreased in the cells co-cultured with Fer-1, showing a decreasing trend (**p < 0.05) (Fig. 2.2.A&C).

**Fig. 2.3:**
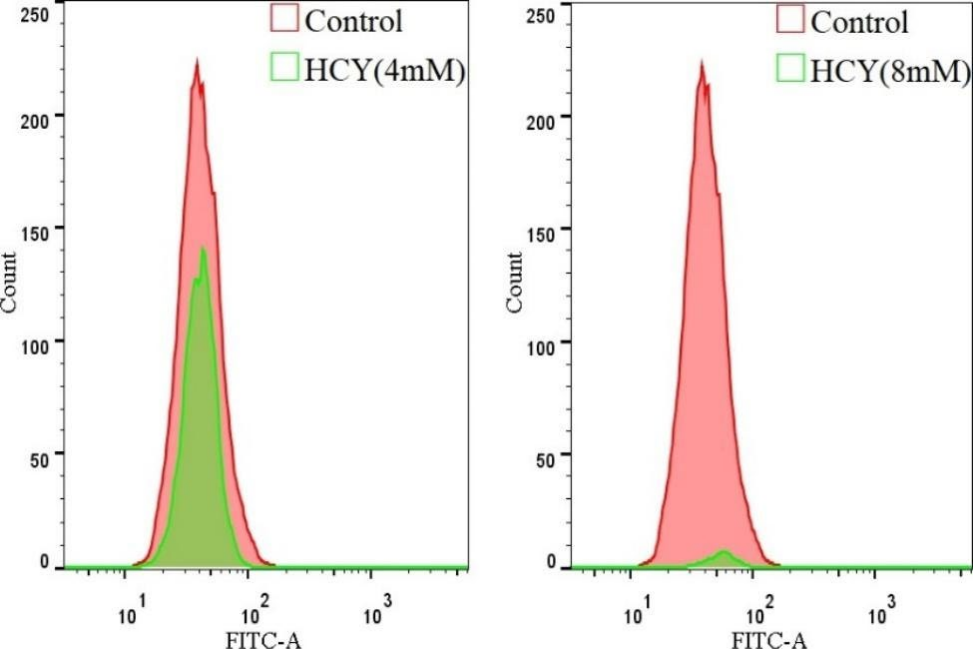
MDA content in EA. hy926 cells after HCY treatment(‾x±s, n = 3) * denotes *p <* 0.05 compared with control group; ** denotes *p <* 0.05 compared with HCY (4 mM) group

Compared with the control group, the content of intracellular MDA increased after HCY treatment and was concentration-dependent with HCY (*p < 0.05), while Fer-1, an ferroptosis inhibitor, could decrease the level of MDA in cells in a concentration-dependent manner (**p < 0.05) (Fig. 2.3). The results confirmed that Fer-1 could inhibit HCY-induced the EA. hy926 cellular lipid peroxidation process.

### Effect of HCY on GSH Content in cells

**Fig. 2.4:**
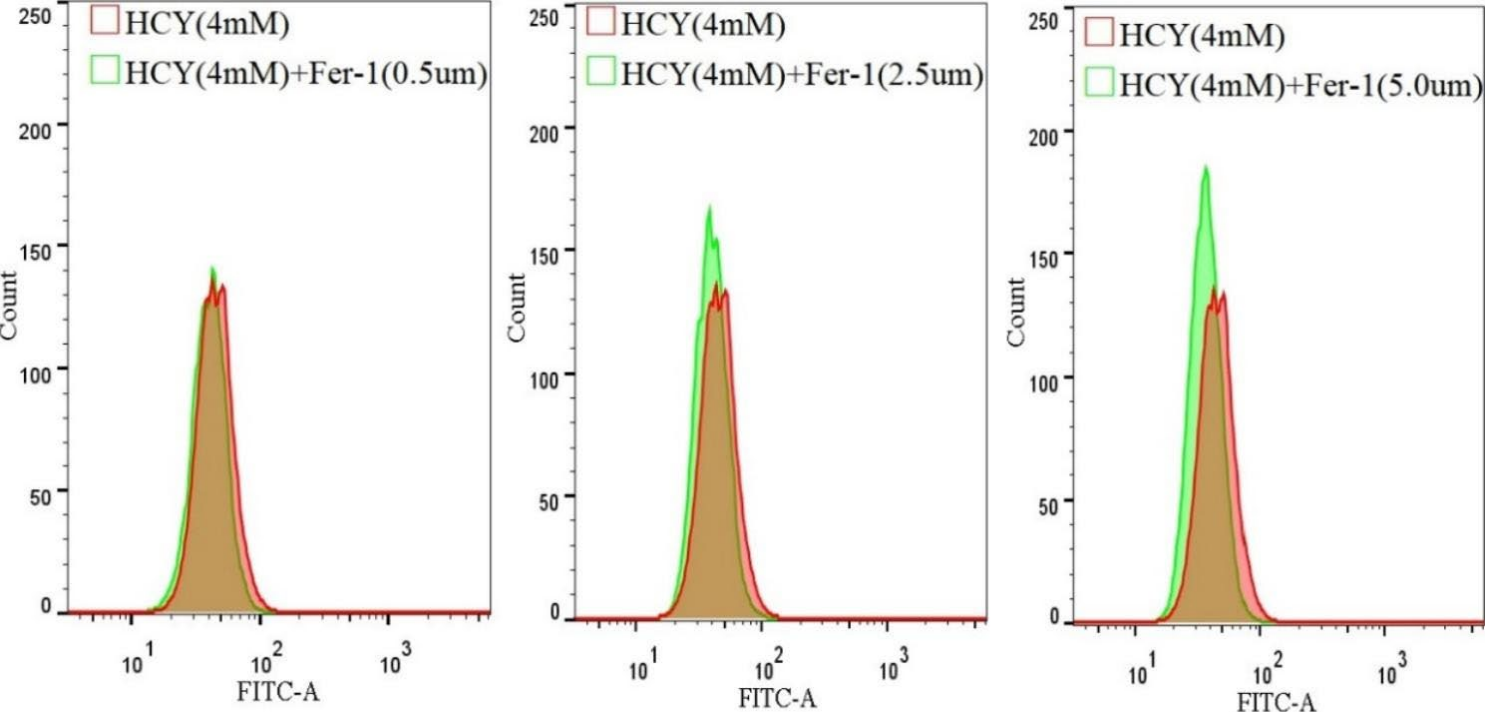
GSH content in EA. Hy926 cells after HCY treatment(‾x±s, n = 3) * denotes *p <* 0.05 compared with control group; ** denotes *p <* 0.05 compared with HCY (4 mM) group

Compared with the control group, the content of GSH in EA. hy926 cells of HCY group decrease in a concentration-dependent manner (*p < 0.05); while Fer-1 could increase intracellular GSH content in a concentration-dependent manner (**p < 0.05) (Fig. 2.4). Showed that Fer-1 alleviated the effect of HCY on oxidative stress damage in EA. hy926 cells.

### HCY induction of cellular ferroptosis-associated protein expression

**Fig. 2.5:**
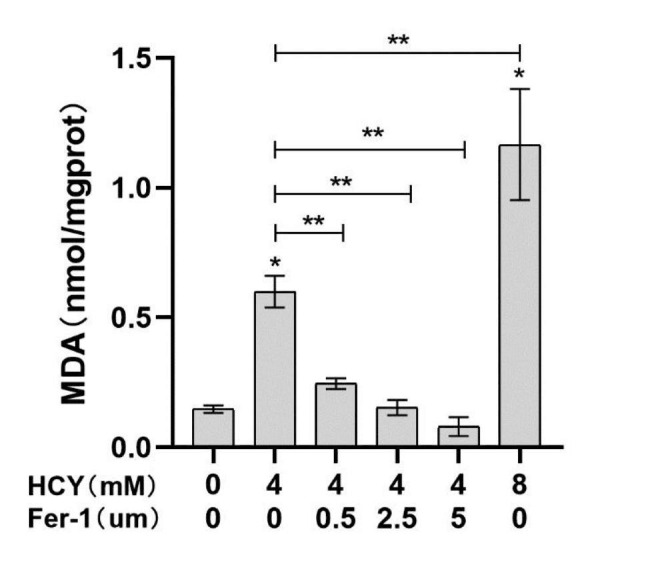
Expression of ferroptosis associated protein SLC7A11 induced by HCY(‾x±s, n = 3)* denotes *p <* 0.05 compared with control group; ** denotes *p <* 0.05 compared with HCY (4 mM) group

**Fig. 2.6:**
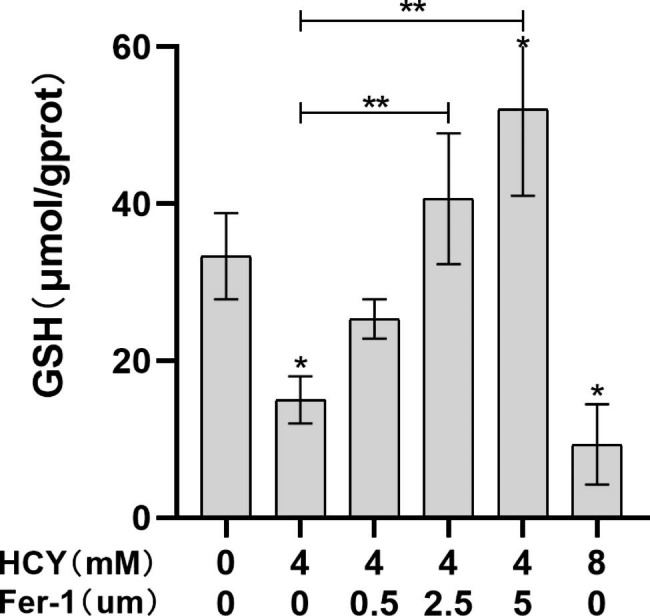
Expression of ferroptosis associated protein GPX4 induced by HCY(‾x±s, n = 3)* denotes *p <* 0.05 compared with control group; ** denotes *p <* 0.05 compared with HCY (4 mM) group

Compared with the control group, the expression levels of GPX4 and SLC7A11 protein in the HCY group were significantly decreased (*p < 0.05), and the up-regulation of GPX4 and SLC7A11 protein was significantly promoted after Fer-1 co-culture and the protein expression levels could increase with the increase of Fer-1 concentration (**p < 0.05) (Fig. 2.5&2.6).

## Discussion

A large number of studies have confirmed that improving vascular endothelial cell function and protecting endothelial structural stability is particularly important for the prevention and treatment of atherosclerotic diseases. Factors such as vascular oxidative stress, vascular inflammatory response, reduced NO bioactivity, vascular aging and hemodynamic changes can cause endothelial dysfunction and then lead to cell death.

Recent studies have found that endothelial cell injury can induce a novel form of death, ferroptosis, in addition to initiating programmed death forms such as apoptosis, necrosis, and autophagy. Bai [5] found key features of ferroptosis such as increased lipid peroxidation products, iron accumulation, and decreased expression of glutathione antioxidant core enzyme GPX4 in a mouse model of atherosclerosis induced by iron, while ferrostatin-1, an ferroptosis inhibitor, significantly reduced arteriosclerotic plaque area after the intervention; similarly, Xiao [6] found decreased cytoplasm of death, shrinkage of intracytoplasmic mitochondria, and specific changes such as lipid peroxidation, iron accumulation, and decreased expression of ferroptosis-related protein GPX4 when stimulating ferroptosis in umbilical vein endothelial cells using the small molecule ferroptosis inducer Erastin, indicating that ferroptosis does exist in endothelial cell injury in the early stage of atherosclerosis. Therefore, an in-depth study of the effect of ferroptosis on the pathophysiological process of atherosclerosis is significant and the basis and purpose of this experimental design.

Homocysteine is an important substance for intravascular metabolism and is closely related to the occurrence of cardiovascular disease as a factor of atherosclerosis. Elevated blood homocysteine levels can affect vascular endothelial function and lipid metabolism, promoting atherosclerotic plaque formation and thrombotic events [7]. Homocysteine can cause endothelial cell oxidative stress, lipoprotein metabolism disorders and other endothelial cell dysfunction, leading to the production of a large number of reactive oxygen species and vascular inflammatory factors in cells to inhibit the normal diastolic function of blood vessels and thus promote the progression of atherosclerotic lesions. In addition, homocysteine can inhibit the activity of the expression of antioxidant substances in endothelial cells such as superoxide dismutase, glutathione peroxidase and heme oxygenase, indicating that homocysteine can simultaneously promote oxidative stress and inhibit the function of the antioxidant system to damage vascular endothelial cells causing apoptosis and necrosis [8, 9]. Recently, it has been found that homocysteine can also mediate ferroptosis. Zhang [10] found in a homocysteine-induced disc degeneration model that homocysteine accelerates the progression of spinal degenerative changes by inducing cellular ferroptosis in nucleus pulposus cells. Early studies have found that there are specific changes in ferroptosis such as lipid peroxidation and iron ion accumulation in the process of atherosclerosis, and homocysteine has also been shown to enhance cellular lipid peroxidation, but the existing studies have not explored it in depth. According to the results of this experiment, after homocysteine stimulation, the viability of EA. hy926 cells were significantly reduced, and the contents of Lip-ROS and MDA, products of lipid peroxidation, were significantly increased, while homocysteine-induced endothelial cell lipid peroxidation was significantly improved after application of Fer-1, a specific inhibitor of ferroptosis, and the stimulation and inhibition processes were concentration-dependent, preliminarily demonstrating that ferroptosis is involved in the process of homocysteine-induced endothelial injury.

GPX4, the core regulator of ferroptosis, is the only enzyme known to reduce phospholipid hydrogen peroxide, which can convert intracellular toxic lipid hydrogen peroxide (L-OOH) into non-toxic lipid alcohols (L-OH) to remove harmful substances such as superoxide and block the occurrence of chain peroxidation, and is one of the key proteins for ferroptosis. Yang [11] found that the sensitivity of cells to ferroptosis inducers was increased after overexpression of the GPX4 gene, and conversely, knockdown of protein expression of GPX4 promoted cells to undergo ferroptosis. GSH is an essential antioxidant substance in the process of oxidative stress defense. Its intracellular and extracellular transport and synthesis mainly depend on the regulation of systemXc^−^ on the cell membrane surface. systemXc^−^ can mediate the reverse transport of extracellular cystine and intracellular glutamate. On the one hand, it ensures the adequacy of intracellular GSH content, and on the other hand, it can reduce the toxic effect of intracellular glutamate on cells. Since GSH is an essential cofactor for GPX4 to function, systemXc^−^, as an upstream regulatory protein of GPX4, can affect the activity and expression of GPX4 and is also an important regulator in the ferroptosis pathway [12]. In this study, we further investigated the possible mechanistic pathways when the homocysteine-induced endothelial injury occurs in ferroptosis by analyzing the protein levels of ferroptosis-related proteins GPX4 and SLC7A11. The results showed that homocysteine promoted endothelial cell lipid peroxidation, aggravated endothelial cell oxidative stress, and significantly down-regulated the protein expression of GPX4, a key protein of ferroptosis, in a concentration-dependent manner; meanwhile, we set up three concentrations of Fer-1, a specific inhibitor of ferroptosis, for rescue by referring to a large number of literature, and found that Fer-1 could reverse the ferroptosis effect induced by homocysteine in endothelial cells in a concentration-dependent manner. In addition, a large number of experiments have shown that homocysteine can cause dysregulation of the glutathione antioxidant system leading to reduced GSH content by inducing oxidative stress, so we speculated whether the reduced GSH content in endothelial cells after homocysteine stimulation is related to the ferroptosis-related protein systemXc^−^. In the experimental results, we found that the content of GSH and the expression of systemXc^−^, an ferroptosis-related protein related to GSH transport synthesis, were reduced in cells after homocysteine treatment, and the decreasing trend was consistent, indicating that the decrease in GSH level may be due to the decrease in systemXc^−^ expression which in turn hinders the synthesis of GSH, further confirming the promoting effect of homocysteine on ferroptosis in endothelial cells. Based on the above experimental results, we speculated that homocysteine may induce ferroptosis in endothelial cells through the systemXc^−^/GPX4 axis.

In addition to the classical Xct-GPX4 signaling pathway, the iron metabolism regulation pathway, lipid metabolism regulation pathway, and sulfur transfer pathway can also mediate the occurrence of ferroptosis. The regulation of intracellular iron homeostasis is mainly regulated by transferrin receptors, iron pump proteins, and intracellular ferritin on the cell membrane surface [13]. Pathological damage such as stress can cause a rapid increase in intracellular iron levels, and cells can store part of the excess iron ions in the cytoplasm as ferritin through autoregulation, and the other part is transported by iron pump proteins on the cell membrane into the blood for redistribution. When cellular iron homeostasis loses balance, it can cause intracellular iron overload and then destroy mitochondria, proteins, nucleic acids, etc., resulting in cell damage. Yang [14] found in the experiment that Erastin can down-regulate ferritin light/heavy chain protein expression, promote ferritin degradation to release a large amount of free iron, while up-regulating transferrin receptor levels, so that the cellular uptake of iron is increased, resulting in intracellular iron excess and oxidation due to its easily oxidized characteristics to generate a large number of reactive oxygen species to damage the cells. At the same time, studies have found that excessive free iron in myocardial cells can inhibit calcium influx, affect myocardial excitation-contraction coupling and then cause heart failure, cardiomyopathy and so on [15]. Polyunsaturated fatty acids (PUFAs) on the surface of the cell membrane can maintain the stability of membrane protein structure and function and affect the fluidity of the cell membrane. Due to the instability of its structure, it is easy to be oxidized and cleaved to form toxic metabolites such as MDA and 4-HNE, resulting in changes in cell membrane permeability. ACSL4 and LPCAT3 are important regulators in the process of lipid metabolism and are involved in the anabolism of phospholipids containing polyunsaturated fatty acids. Dixon [16] found that the expression of ferroptosis was reduced after down-regulation of ACSL4 and LPCAT3 genes during ferroptosis induced by small molecules RSL3 and ML162, indicating that ferroptosis can be inhibited by knocking out ACSL4 and LPCAT3 and down-regulating their gene expression to reduce the membrane load of oxidation-sensitive PUAFs. The results of this experiment preliminarily verify the conjecture that homocysteine may initiate the ferroptosis pathway in endothelial cells through the systemXc^−^/GPX4 axis, but due to the limitation of time this experiment cannot determine which node of this pathway is mainly involved in the induction of ferroptosis by homocysteine and whether other pathways are also associated. Therefore, supplementary experiments should be performed subsequently to verify, for example, whether homocysteine will change the expression level of other ferroptosis-related factors after up-regulating or down-regulating the expression of one of the nodal proteins in this pathway by transfection, and increasing the detection of intracellular iron ion content to further verify the degree of ferroptosis during homocysteine-induced endothelial injury.

## Electronic supplementary material

Below is the link to the electronic supplementary material.

**Additional File 1:** Western Blot Original Image

## Data Availability

The datasets used or analysed during the current study are available from the corresponding author on reasonable request.
