# Correction to: Homocysteine induces ferroptosis in endothelial cells through the systemXc−/GPX4 signaling pathway

**DOI:** 10.1186/s12872-023-03384-8

**Published:** 2023-07-17

**Authors:** Jiahao Shi, Di Chen, Zilin Wang, Shaolin Li, Shuying Zhang

**Affiliations:** Zhongshan Hospital of Dalian University, Dalian, China

Following publication of the original article [1], in this article, the order that the authors appeared in the author list was incorrect. It should read as follow:

Jiahao Shi, Di Chen, Zilin Wang, Shaolin Li and Shuying Zhang

The original article has been corrected.
