# Supplementary figures and images for: Homocysteine induces ferroptosis in endothelial cells through the systemXc−/GPX4 signaling pathway
Source: BMC Cardiovasc Disord. 2023 Jun 24;23:316. doi: 10.1186/s12872-023-03342-4 (PMC10290364; doi:10.1186/s12872-023-03342-4)

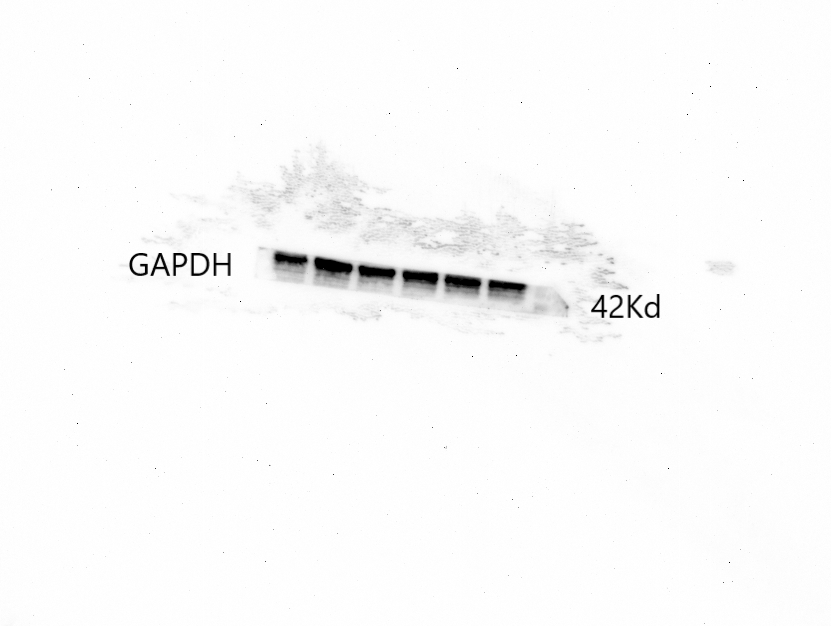

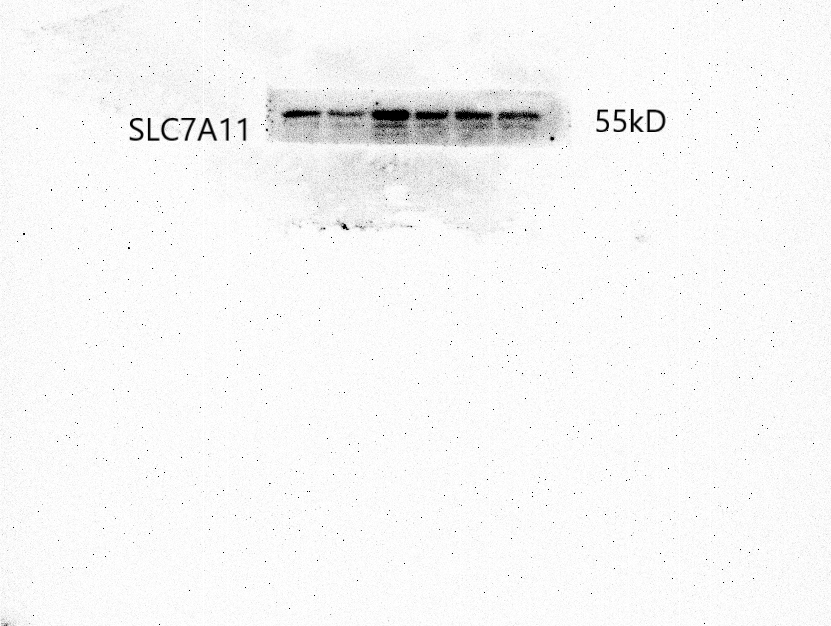

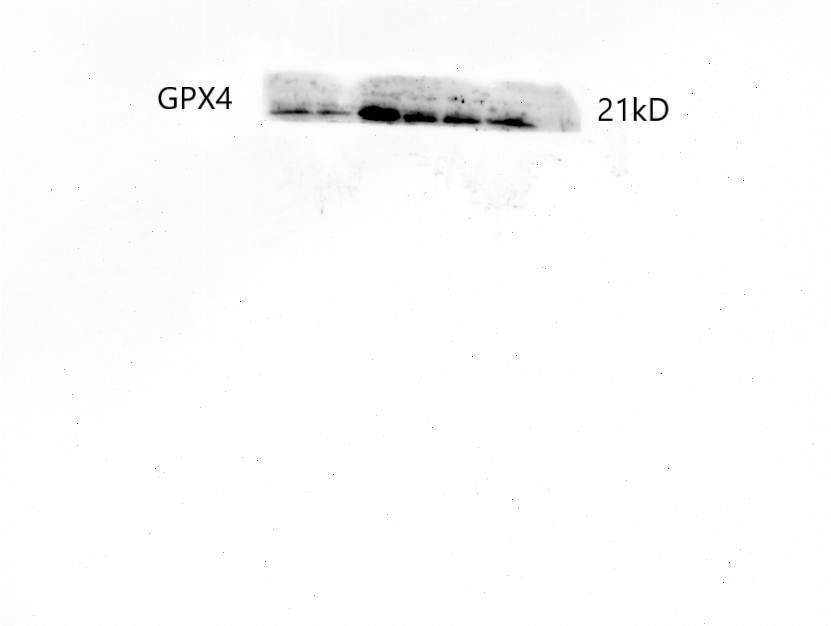

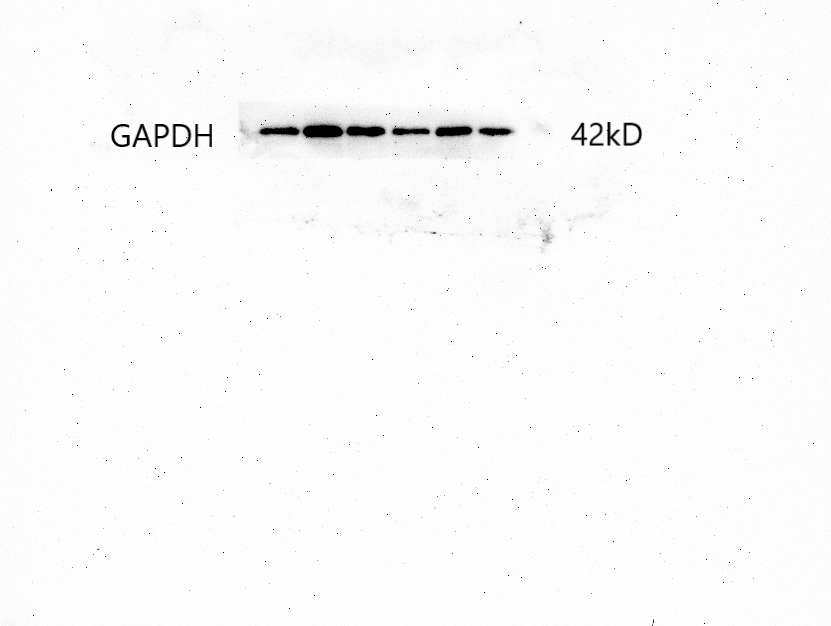

Supplement: Supplementary file 1 — Additional File 1: Western Blot Original Image [file 12872_2023_3342_MOESM1_ESM.docx]
